# The American Dental Association

**Published:** 1894-10

**Authors:** J. M. Walker


### The American Dental Association.

Thirty-fourth Annual Session, at Old Point Comfort, Va., August 7th, 1894.

Reported for the DENTAL REGISTER by Mrs. J. M. Walker.

The American Dental Association convened in thirty-fourth annual session at Old Point Comfort, Va., August 7th, 1894.

The meeting was opened with prayer by the Rev. H. L. Cheatham, Hampton, Va. After an eloquent address of welcome on the part of the Virginia State Dental Association, and the transaction of some routine business, Dr. J. D. Patterson, Kansas City, delivered his annual address. In his address Dr. Patterson paid a touching tribute to the memory of the many members of the association who have passed away during the past two years. He then turned his attention to a number of suggestions looking to increased usefulness of the association. He lamented the "undigested science" and "trivial repetition" which burden the records of the society instead of original scientific matter, the result of diligent labor and thorough investigation. He deplored the lack of self-sacrifice on the part of the many, based on the mistaken idea that to but few is given individual power; but, he said, this is given to all who really work for it. There is no department of science in which investigation will not reward the individual; not half-learning, not superficial science—this brings but ephemeral reward.

The historic method of working by "sections" fails, because so few members of the section assist in the work assigned. So long as the work is voluntary, each one waits for the others, and the result is that the work is all done by a few enthusiastic members and new talent lies undiscovered. If each one would realize that his membership obligates him to make some report, be it much or little, strength would be developed which would give a new impetus to the American Dental Association. The proposed revision of the constitution offers the opportunity of incorporating valuable new features. Among others, that of a "supervisory committee of work" with clearly defined duties looking to more perfect scientific results. Another would be the reservation of the association proper for scientific work, leaving all business routine out of the regular sessions. In a brief review of the allied forces in dental education, the colleges, professional literature, dental legislation, etc., he noted memorable activity and improvement during the past two years. He hoped that some plan would be formulated for the unification of dental laws in the different States so that a certificate of registration in one State would be recognized in any other. He pronounced the present status of dental legislation a travesty on justice and equity.

He recognized the work of the World's Columbian Dental Congress as a prominent factor in the activity of the past two years and rendered due meed of praise to those whose labor of love has been so richly rewarded. He regretted the great increase of unethical practitioners as a drag on professional purity, reputation and advancement. He characterized them as the "anarchists" of our calling. No line too strict can be drawn in this direction; we must permit no infringement upon the strictest letter and spirit of the code of ethics.

The president's address was referred to a committee of three: Drs. Abbott, Crenshaw and Brophy, whose report at a subsequent session, Wednesday, 8 P. M., placed the prominent points of the address before the association, resulting in an interesting discussion. Their consideration of certain points in the address led them to suggest (1) that in the revision of the constitution, clinics in surgical and prosthetic dentistry be re-established, though this step was not directly suggested in the address. After some discussion *pro and con*, Dr. C. N. Pierce said that it was well known that clinics had not been abandoned by the American Dental Association except after very serious consideration. He moved that the matter be referred to the executive committee "with power to act."

DR. THOS. FILLEBROWN pronounced this a cowardly way of meeting the question. As a matter of fact, the association either wants or does not want clinics. He moved that the matter be laid on the table indefinitely. This motion being duly recorded, was carried.

2. That the suggestions in regard to registration certificates of one State being made valid in another State, without further examinations, were impracticable so long as the members of the Examining Boards were selected in so many different ways. On motion of Dr. B. Holly Smith, a committee of three, Dr. B. H. Smith, A. W. Harlan, J. Y. Crawford, were appointed to confer with similar committees from other dental associations, national, State or local, in an effort to secure unification of dental laws in this country.

3. Various methods were suggested of securing more efficient work in the sections, especially something from each member. The most feasible idea was that suggested by Dr. Weeks, of Minneapolis, that the chairman of the section make a specific request of each member of the section. It would of necessity be optional with each one to answer or to refuse to answer, but the various replies received, properly edited by the chairman, would in itself furnish a special report!

Other portions of the address were referred to the Committee on Revision of Constitution and By-laws, and the subject was passed.

The subject of the coming celebration by the dental profession of America, of the fiftieth anniversary of the discovery of anæsthesia, by Horace Wells, of Hartford, Conn., was introduced by Dr. J. V. Thomas. He mentioned the steps taken by the Odontological Society of Philadelphia, but said that the subject was one of national importance, and too great for local societies to handle. On his motion a committee of nine as follows: Thomas, Fillebrown, Taft, McManus, W. H. Morgan, W. C. Barrett, Harlan, McKellops and Weeks was appointed to consider the matter and report to the association.

Dr. McManus called attention to an article on this subject recently published in the *Century Magazine* as being not only misleading, but as containing statements that are absolutely false, and desired that the association re-affirm the resolutions adopted in 1864, based on the results of an official investigation by a congressional committee. (The facts in the case are contained in a work on modern anæsthesia published in 1867, by the late H. F. Smith, United States Senator from Connecticut.)

Dr. Brophy suggested that in any resolutions adopted by the association, the words "United States of America" be omitted, as this is not a local affair. The benefits of modern anæsthesia extend over the whole world.

Dr. Harlan thought it essential to insert explicitly the date of the publication of the results of the legal investigation that fully established the claims of Dr. Horace Wells; to fix the time and place, when and where the investigation was held, and where the documents are published that give the results of this legal investigation.

At a later session the committee reported a series of resolutions and a partial programme for the celebration, which will be published when all arrangements are perfected.

On motion of Dr. H. A. Smith, Dr. J. N. Crouse made a report on the present status of the litigation being conducted by the *Dental Protective Association.* He said that the past year had been one of special activity in securing testimony in the Low Bridge Case, testimony having been taken in Denver and even in California. The testimony is all in now, and the case will be argued in October, and then the result of all this labor will be made known. He considers that there is abundant evidence in to defeat the Low Patent in any Court. Dr. Crouse requested that a committee of three be appointed to meet a similar committee of two from the Southern Dental Association, to examine the books of the Dental Protective Association, papers and vouchers, notes and mortgages, and pronounce whether the moneys of the association had been honestly and economically used. The available funds of the association were about $3,000 apart from a mortgage note of $10,000 on real estate, on which it would not be possible to realize at present without great sacrifice. He did not know what the lawyer's fees in the present case would be, but possibly it might be necessary to impose an assessment on the membership rather than touch the mortgage fund. The chair appointed Drs. H. A. Smith, C. E. Esterly and Louis Jack, on this committee—Drs. R. R. Freeman and V. E. Turner having been appointed by the Southern Dental Association. At a later session the committee reported that they had examined the books and papers of the Dental Protective Association and found everything correct and satisfactory. On motion the report of the committee was received and approved.

The resignation of Dr. M. G. Jenison, Minneapolis, was received and accepted.

At the night session Tuesday, on motion of Dr. W. W. Walker, Dr. John S. Marshall, Chicago, as treasurer, offered a report from the executive committee of the World's Columbian Dental Congress showing the present financial status. This statement embraced a detailed account of all moneys received of the treasurer from foreign countries and from the different States and State Societies of the United States, making a grand total of $13,790.05, not including $400.00 in the Columbian National Bank at the time of its failure, from which dividends amounting to $249.74 have been received, making the sum of $14,309.79 received. The disbursements have somewhat exceeded this sum, and by the time the bills for publishing the Transactions, including contracts for engraving illustrations, etc., there will be a probable deficit of $1,000.00. The above statement does not include the expenses of the executive committee for railroad and hotel bills, etc., amounting in all to some $4,000.00 which has been cheerfully donated to the Congress. It is desirable however that the sum of $1,000.00 be raised to pay for the Transactions, that they may be distributed to those entitled to them as early as possible. Various methods of covering the deficit were suggested. Finally, on motion of Dr. H. A. Smith, duly recorded, an appropriation of $500.00 from the funds of the association was voted to the executive committee of the World's Columbian Dental Congress. Dr. Marshall stated that there were three hundred of the memorial medals still on hand and for sale at $10.00 each. Also extra copies of the Transactions could be secured, and he hoped by these or other means to raise the other $500.00.

At a later session, Dr. Marshall announced that he had raised $260.00 and hoped to complete the sum of $500.00 before the close of the meeting. The Transactions had to be paid for and it was not right to allow the members of the executive committee of the Congress to pay the bill.

A meeting of the National School of Dental Technics was announced for 3 P. M., August 8th, Dr. J. C. Stephan, Secretary.

SECTION 1. PROSTHETIC DENTISTRY was then called.

DR. W. E. SHARP, Binghamton, exhibited and explained a new furnace for general laboratory purposes—continuous gum, porcelain, aluminum and other metal work, etc., which apparently offers many advantages over the other furnaces for similar work now on the market. As now manufactured it is only adapted to the use of gas as fuel (with No. 9 A, Buffalo foot-blower), but will probably be adapted to the consumption of gasoline, coal oil, etc. In reply to questions it was stated that it fully replaces the coke furnace for continuous gum-work; heating up is done very rapidly; there is no lost heat, but the heat can be perfectly regulated to suit the work in hand whether for low-fusing enamel, aluminum casting, or any work for which a furnace is required.

DR. GEO. EVANS, New York, demonstrated his method of constructing hollow gold dummies for bridge-work. The band and cusps being completed, a little ball of fluxed solder is placed on a piece of platinum, 30 or 32 gauge, over which the hollow crown is placed. Heat being applied beneath the platinum, the solder flows to the edges all around on the inside. The edges of the platinum are then trimmed and stoned down forming a hermetically-sealed, hollow dummy.

Dr. Evans also demonstrated a method of crowning front teeth, the root being banded on the approximal and palatine sides with no gold showing at the labial surface. A peculiar feature of the method is the means by which the pin is securely fixed in the cap. By means of a suitable die a small counter-sunk spot is formed in one end of a long oval disk, the opening of the root canal being reamed out to receive this depressed portion of the cap—pure gold is melted into the concave space, through which the pin is screwed, being held firmly in place by the thickness of gold in the depressed portion. The disk and pin is then adjusted to the abutment of the root and trimmed even with the labial portion, the projecting portions at the approximal and palatine sides being snipped and turned back on to the root, removing and annealing as necessary. The porcelain tooth being backed as usual and soldered to the cap in such a manner as to secure perfect continuity of all sections of the crown, giving all the strength of the regular crown with no metal appearing at the cervico-labial margin, while simplicity of construction is promoted. The crown is secured to the root with oxyphosphate, but a special feature in Dr. Evans' method is that a film of gutta percha is put on the pin before inserting it in the cement in the root canal, so that in case of accident it is easily removed, heating the crown in the mouth with a suitable instrument, softening the gutta percha on the pin so that it is readily withdrawn leaving the cement in the root canal to be reamed out for re-insertion of the crown. Dr. Evans' methods were discussed by Drs. Freeman, Sill, Crouse, Gardiner, Brophy and others, many questions being asked and answered.

Dr. Brophy said: All this leads us back to the days when we had *clinics*. Such methods as these cannot be clearly and satisfactorily explained without an ocular demonstration. Telling a person how to do such things is never as satisfactory as showing him how it is actually done. I hope the time will come when clinics will be re-established in the American Dental Association. Every one would be glad to see the methods of a man like Dr. Evans practically demonstrated.

Dr. H. B. Noble presented Dr. Daly's method of lining vulcanite plates with an indestructible lining of pure gold—a lining which lasts as long as the plate itself and which gives a smooth, even, polished metal surface next to the mucous membrane of the mouth. The lining is produced by depositing crystals of pure gold on one side of a sheet of No. 20 gold foil 'till it attains the grade of No. 50 or 60 foil. The lining material thus has a smooth, polished, bright gold surface on one side and a roughened crystalline surface on the other. To apply it, after the flask has been opened and the wax removed, the cast is varnished with sandarach varnish and dried. A coating of damar varnish is then applied to give a sticky surface so that the lining material will not slip or slide out of position. The lining is cut in small pieces varying from one-fourth to one-half inch, varying in shape to suit the surface to be covered. The pieces are laid on with the bright side down and overlapping so that there will be no cracks or uncovered portions, every portion of the surface being perfectly covered with the lining. The overlapping of the small pieces is an important feature in adjusting the lining to the cast. The rubber is then placed as usual, being careful not to have it thicker than necessary, and of uniform thickness throughout. Vulcanize the piece as usual. In finishing up the piece, burnish and brush the gold surface fearlessly. It cannot be rubbed off. The vulcanite piece thus lined has a solid gold surface and is as cleanly as an all-gold plate. The rubber thus vulcanized on the gold lining is more dense than by any other method.

Dr. St. George Elliot described a method of swaging through the force of steam power, the work being done with great rapidity and perfect ease to the operator.

Dr. Custer described a method of swaging plates with shot instead of counter-die, also the process of melting platinum almost instantaneously by means of the incandescent light current.

Dr. Bogue described a hydraulic press for swaging metal plates giving, with very little trouble, a more perfect adaptation to the plaster model than could be secured by the hammer or a pile driver.

Dr. Butler apprehended that it would be found, if carefully tested, that the gold had been thinned to a very considerable extent in places, making the close fit at the expense of the strength of the plate.

Dr. Bogue replied that it was necessary to use judgment in applying the force. One young man burst asunder a ring of malleable iron three-fourths inch in thickness. The subject was discussed and various methods of swaging by mechanical apparatus designed to replace the hand power and sledge hammer of the operator described.

Dr. V. H. Jackson, New York, described, from a series of large-size illustrations (copyrighted), and a number of plaster-casts, his "crib" method of regulating and retaining appliances. In these appliances Dr. Jackson uses German silver wire, 14

gauge, using soft solder unless there is great acidity of the oral fluids, or the fixture is to remain long in place. If a permanent fixture, to hold in position teeth loosened by pyorrhœa alveolaris, he uses iridio-gold or iridio-platinum wire. For a strong spring wire he uses piano wire tinned, if it is to remain in the mouth for some time. The crib-springs are of German silver wire slightly larger than the piano wire used. A number of special cases with the fixtures used were described as illustrated by the diagrams exhibited, which are essential to an understanding of the method.

Dr. J. Y. Crawford asked Dr. Jackson what he thought of the propriety of removing very loose teeth, and after the removal, of all deposits, replacing the teeth in the mouth.

Dr. Jackson replied that a tooth which had been affected by calcareous deposits was not a fit tooth for replantation. A replanted tooth if held at all was held by granules of living tissue penetrating the interstices of the tooth substance. Calcareous deposits on the teeth infiltrate these spaces, while the roughened surface left after scraping off the deposits creates an inflammatory condition in the surrounding tissues and the tooth is thrown off. No artificial means can restore the smoothness of surface nature requires.

Dr. W. H. Morgan rose to a point of order—the discussion was invading the field of pathology, which will be discussed in connection with other papers. Dr. Morgan asked Dr. Jackson what was meant by the term "spring gold?"

Dr. Jackson: Simply what is sold by all dealers as *spring gold*, I suppose it is platinized gold.

Dr. Morgan: The best spring gold is made by the addition of one-tenth part of platinum added to pure gold. If two parts platinum is added to the ounce of 22 or 23 carat gold, it makes a very good spring gold.

Dr. Jackson: A very important thing would be a non-corrodible metal with the spring of steel; a metal that could be heated many times and yet retain its spring. I have an experienced metallurgist experimenting in this direction and hope to get it yet.

Dr. W. O. Kulp explained a method of retaining in place loose incisor teeth with extensive absorption of the alveolus. His method differs from that of Dr. Jackson in that the entire lingual surface of the teeth is covered with gold bands which extend under the free margin of the gum, being cut nearly down to the gum line at the labial surfaces of the teeth. The portion on the labial surface may be of platinum, which is less offensive to the sight than gold. The bands are all soldered together in the lingual side making a continuous metal surface, securing the loose teeth so firmly that they have been retained in the mouth for twenty years after they were so loose that it was difficult to keep them in place while adjusting the bands. He had utilized the same idea in case of a fracture in the center of the lower jaw, soldering a plate to the bands after adjusting the fracture. The day after this fixture was adjusted the patient came to him saying: "Thank God; I was able to eat a beefsteak for my breakfast." A regular surgeon predicted at the time that there would be no osseous union, but six months later the case was reported a perfect success.

Dr. J. N. Crouse described an automatic pump, worked by water pressure, for condensing air in a cylinder giving a current of air, or a blast sufficient for any purpose desired, whether for continuous-gum furnace, or for soldering. He has it connected by piping up to his laboratory table when he has simply to open a stop-cock to get any amount of air wanted. They are too high-priced at present but he hoped to be able to handle them soon at $15.00.

Dr. W. O. Kulp said that Professor Hunt, taking the idea from the beer pump, had invented an air pump to be attached to the water pipe which only costs four or five dollars and has sufficient force for the continuous-gum furnace.

Section 2. Dr. L. Ottofy, chairman, "Dental Education, Literature and Nomenclature," was next called.

Dr. Ottofy reported first on the number of dental colleges. At the time of the last report from this section in 1892, there were thirty-nine. This number has been augmented to forty-six in two years, two of which, however, have been discontinued, leaving forty-four dental colleges in actual operation. Two thousand eight hundred students have matriculated, of whom two thousand six hundred have been in attendance and six hundred have been graduated.

The National Association of Dental Faculties has been the source of marked development and advancement in the standard of the colleges, and but one dental college, as far as known, now continues the traffic in dental diplomas. Necessarily all do not have the best possible equipment or the best of teachers, but all recognize that they cannot attain to prominence or maintain their standing unless they enforce the rules laid down by the National Association of Dental Faculties. Credentials from foreign schools are scrutinized with the utmost caution and the rule is enforced that foreign students must acquire our language.

The subject of dental education was exhaustively treated at the Columbian Dental Congress and at the Twent-first International Medical Congress in Rome, and many points were raised that are worthy of investigation.

In handling the large number of students in some of the colleges by sections and sub-sections, it is impossible that full justice should be done to each and every individual. In the extracting rooms a student has perhaps but one or two days' actual practice. The subject of either limiting the classes or enlarging facilities is one of great importance. A student may well ask if it was the right thing to admit him when it was not possible to furnish him with clinical material. It has been suggested that students be given the privilege of practical work under the tutelage of competent practitioners, but this is not always feasible. The custom of changing from one school to another, during a student's course, is to be condemned, as schedules are not uniform and some points may be lost entirely. It is best to select a good school to begin with and to finish there. As to the question of undergraduates being permitted to practice between sessions, this is an evil that is hard to contend with and that is not within the control of the colleges, but the schools should exert a wholesome influence in this direction. The schools should demand that a student, before matriculating, sign the code of ethics; this would exert a great restraining influence. It would be well if every student, before matriculating, should have to sign a statement that he had not been practicing in violation of the code of ethics for two years before matriculation; that he had not been engaged in any office so conducted, and had not had any interest, financial or otherwise in any office, institution, or practice of that nature, and that he had not violated the dental law of any State during that period, and, *secondly*, that he agrees from the day of matriculation to the day of graduation to govern his conduct by the code of dental ethics, to obey the dental laws of his State and not to engage in unlawful practice, and that any violation of this contract would entitle the college to remove his name from its records and to so report to other colleges, and *thirdly*, that a diploma shall be held only during good behavior; that the college have the right to cancel it for any unethical professional conduct. All this to be signed in due legal form. Such a contract as this would draw a much better element to our colleges and would exert a most wholesome influence on the whole dental fabric.

The report considers this a most propitious time to introduce improvements and reforms tending to elevate professional standing. The first vital demand is a change in the qualifications for admission to a dental school. To allow each school to be the judge of the individual fitness of the candidate is a faulty method. The lowest requisite should be the degree of Bachelor of Art or its equivalent obtained from a reputable university. The school term should also be extended to nine months each, for four years.

The national organization of the teachers of technics is a very important step. This feature, introduced by Dr. G. V. Black, is most important in the education of the dental student.

The system of post-graduate instruction has proved very beneficial in improving the older practitioners who had not the advantages of college education and in improving graduates in special branches. It is greatly to be regretted that the adoption of a uniform system of dental nomenclature did not result, as was hoped for, from the World's Columbian Dental Congress. A better means of popular education is an imperative demand. This should be parcelled out in small doses, by means of slips adapted to the case in hand, giving just the information needed, but not trying to teach it all at once. The section had hoped to submit reports from many local societies, and as a sample of what was desired, submitted a report from the Washington City Dental Society as a model.

In the discussion of the topics introduced in this report–

Dr. Frank Abbott thought that the proper place for reform, in the time devoted to dental education, was at the beginning rather than at the end of the term. He was in favor of raising the requirements for admission to the school rather than extending the time for technical study.

Dr. St. George Elliot said that in education *per se*, England was decidedly ahead, though in technical skill the American dentist was far ahead. He thought Dr. Abbott had hit the nail on the head in what he had said as to the necessity of more thorough preliminary education and a higher standard of admission to the technical schools.

Dr. W. H. Morgan said that success in the practice of dental surgery depends largely upon technical skill. You may give a man all the collateral science you choose, but without technical skill he will not be a competent dentist.

Dr. Frank Abbott: The fact of a thorough preliminary education does not preclude the possession of practical skill. The better a man is educated the better professional man will he make.

Dr. Bogue called attention to the fact that in Austria a man had to complete a seven years' course in medicine before he could take up dentistry, and for that reason there were no good dentists in Austria. A man needs to acquire his technical skill while he is acquiring his theoretical education.

In Italy no man can study medicine till he has graduated in literature, and he cannot study dentistry till he has graduated in medicine. He must take the degree of A.B. before he can get the M.D., and he must take the M.D. before he can get the D.D.S. Time is not counted in the old countries and a man would be forty years old before he was prepared to do anything in dentistry, but here every moment counts. A young man begins at eighteen and in three years he expects to begin to do something for himself. He cannot wait till he is thirty or forty years old before he begins to serve his fellow men. At eighteen or twenty he is prepared to enter a professional college and at twenty-one, two or three he is ready to earn his living and to render good service in his chosen profession.

Dr. J. N. Crouse: In regard to practicing before graduating, I think every man should be given a chance to find out what he is fitted for as early as possible, and if he is not fit for it let him find it out early and try something else. A man does not want to spend three or four years in learning something he is not adapted to. The college faculties know that many of their students will never make dentists. Then let them try and find it out, so that they can quit in time and go at something else.

Dr. J. Taft: That would perhaps be an advantage to the undergraduate and not hurt him, but how about his patients? It takes but a few years to complete the curriculum and before it is completed he is deficient in many lines, especially when a graded course is followed. A man should be as thoroughly equipped as possible before he is allowed to practice at all. He has the opportunity in a well regulated college to test his abilities under competent supervision and direction. It is wrong to encourage the idea of an undergradute starting out alone. He may attain a moderate degree of success—some people are easily satisfied—and then the chances are that he will never complete his course, but will spend the rest of his time making money and believing that he is doing as well as others. A properly planned course should be followed to the end. Those in charge should do their best to give him the best possible equipment before they sanction his going out to practice. The influence of the contrary practice is prejudicial to the profession.

Dr. Morgan: I admit that the broader and more thorough is man's education—all things being equal—the better qualified he is to serve those under his charge. But after all, manipulative ability is the keystone in the arch of dentistry. We cannot do without that. One point in the paper I must refer to—the idea that a young man who, by some mishap through lack of advantages or of proper information, has been led into quackish practice, must never be admitted to a dental college. According to my faith there is no sin that cannot be pardoned through repentance and reformation. If the young man finds out his mistake, if he proposes to rectify the error of his ways, we should take him by the hand, encourage him, lift him up out of the slough of despond. Many of our best men picked up their first ideas in the shop of some "Cheap John;" I do not understand what is meant by the phrase *thoroughly qualified.* A man would never begin to practice if he had to wait till he knew it all first. The schools, through their laboratories and clinics, teach all who have any practical ability what they have to do in a successful practice. If not, they fall short of their duty.

Prof. Truman favors preliminary education that enables a man to profit by the teachings in the professional schools, but would not have a man devote all the best years of his life to it. After a certain age he cannot acquire the manipulative ability necessary to make him a good practical dentist. We do not want the standard of Europe in America.

Dr. F. Abbott followed in the same line of thought with Drs. Morgan and Truman. A dentist if not practical is good for nothing. A minister is good for nothing if he cannot preach. A lawyer is good for nothing if he cannot make a speech. A physician is good for nothing if he cannot, once in a while, cure a patient, and so with a dentist, he is good for nothing if he cannot use his hands. In the old countries the system of education is such that a man is old before he begins his professional life, and so it is that in Germany, France, Italy, Austria, their dentists lack that genius of manipulation that the American dentist is endowed with.

Dr. Bogue: The reason for this is that in a republic every man is as good as the best if not a little better; but in Europe a man who labors with his hands is degraded by it. Dr. Bogue said that his own preceptor taught him first of all how to use his fingers and he honored his memory; he was taught in the little material details of every day work. Manual and intellectual training must go hand in hand.

Dr. Taft: Any man belonging to a profession should be fitted to occupy a respectable place in the intellectual world.

More than half of the men who ten years ago were known as good practical dentists were deficient in a knowledge of their native tongue. I know, from my correspondence, that they could not write a respectable page, not a composition on theory or science, but a simple English letter of a dozen lines. I have been made to blush for the ignorance of men who were practicing dentistry; there is no excuse for this. A respectable common school, or even grammar school education can easily be attained before the age of sixteen or seventeen. Young men are coming out now by the thousands at the age of sixteen, with a good degree of attainment. It is within the reach of all and *it should be required.* Because a few have not had these advantages they should not be permitted to weigh down the whole profession. I speak of a reasonable basis of elementary education. I do not include Latin and Greek and the higher mathematics, though the few men who have had these advantages stand head and shoulders above those who have been less favored. The young man who has been trained in mathematics and the languages is the better prepared to understand the special studies of our own curriculum. A knowledge of the languages from which our own has been built enables us better to understand our own in all that pertains to it. It is not simply a matter of dogged memory, it is a knowledge of the principles underlying its structure. Knowledge of this kind will not, can not interfere with a man's technical abilities. But if he follows the plow till he is thirty or forty years old he will never acquire the technical ability required in dentistry. But all have not the same natural skill in finger-craft; some, whether educated or ignorant, take it up rapidly, some are naturally slow and some are quick. Manipulative ability does not depend upon education.

Dr. Jno. S. Marshall: I agree in the main with what Dr. Taft has said, but I go a step farther, a long step farther. It stirs up a feeling of indignation to hear gentlemen on this floor—gentlemen who are members of college faculties—decrying a liberal education as disqualifying men as students of dental science. It seems to me if we would have dentistry on an equal standing with other liberal professions it must be on the basis of scientific attainments. Who are the men who write the text-books we give our students? They are the men who have gone though college, who have then studied medicine, and have then studied dentistry—they are the men who write our text-books! Dr. Taft knows very well that many men who are "good dentists" write papers that have to be very much *fixed up* to fit them for publication. If they had had a liberal education that would not be so, and they would be none the less *good dentists.* It has been said that the colleges should devote more time to practice and less to theory? But the only place to get any theory is in college. A man will not study theory when he gets out of college and gets into practice if he has not studied it in college; but the best practice is that which has theory and science underlying it.

Prof. Truman: My observation does not agree with that of Dr. Taft, that the average dentist is an ignorant man. It is perhaps true of Michigan, but it is not true of the Eastern States. I am daily brought in contact with written papers, written for publication, and I do not find in them any proof of that excessive ignorance spoken of. I find that they compare very favorably with papers written by medical men.

Dr. Taft: I did not say that the average dentist *of to-day* was an ignoramus. I spoke of the average dentist of ten years ago. Improvements are being made all the time, and Dr. Truman is helping to remedy the defects. I admit that there is a large amount of knowledge in the East, but we are trying to get a little in the West also, and I hope that in the course of the next century we will get up with the East!

Dr. Kulp: I have always advocated a higher order of education as essential to the advancement of the profession of dentistry. We must educate in manipulative skill while we are piling it in on the brain.

Dr. T. T. Moore: Not one-third of the men who get through our dental colleges are competent to practice dentistry. It is not the fault of the colleges. With so many colleges there are not enough students of the right stamp to go round. A practical solution of the question would be to have our dental colleges endowed institutions, with good salaries for the professors. Then if they found a student not competent to make a practical dentist, they could dismiss him at once. It is not right to keep a man three years studying for something for which he is entirely unfit. As soon as this is ascertained he should be told so and advised to try something else.

Dr. H. J. McKellops: I was one of the unfortunate ones who had no early advantages. I had to earn the money with which I got my education. I began at the very bottom of the ladder but I made my own way, and I made my mark in the medical college; my specimens are even now to be found in the dissecting room of that college. But how many medical men look for the source of disease in the mouth? How often does the oculist look to see if a diseased tooth is not the cause of the eye trouble? Men have paid five hundred dollars for treatment of ear trouble when it all came from bad teeth. A dentist should be taught fine manipulative work first. Let them start at the foundation. Do not make plasterers and daubers of them, but teach them nice little work at first and they will never forget it.

Dr. B. H. Catching wished to say a word about the negro in dentistry. There is a negro college in Nashville which is sending out negro graduates in dentistry. When they come before the State Boards for license, they show themselves thoroughly up in theory and in the literature of the profession. They pass fine written examinations, but their manipulative powers are defective. One feature not yet alluded to in this discussion is the present elevated social position of the dentist. A few years ago the dentist occupied about the same position socially as the chiropodist, which was due to his general lack of higher education. His present elevated status is due to the liberal education required as a professional man.

Subject passed.

[*To be continued.*]

